# SSEL: spike-based structural entropic learning for spiking graph neural networks

**DOI:** 10.3389/fnins.2025.1687815

**Published:** 2025-11-28

**Authors:** Shuangming Yang, Yuzhu Wu, Badong Chen

**Affiliations:** 1 School of Electrical Automation and Information Engineering, Tianjin University, Tianjin, China; 2 National Key Laboratory of Human-Machine Hybrid Augmented Intelligence, National Engineering Research Center for Visual Information and Applications, Institute of Artificial Intelligence and Robotics, Xi’an Jiaotong University, Xi’an, Shaanxi, China

**Keywords:** spiking neural networks, graph neural networks, structural entropy, neuromorphic computing, brain-inspired intelligence

## Abstract

Spiking Neural Networks (SNNs) offer transformative, event-driven neuromorphic computing with unparalleled energy efficiency, representing a third-generation AI paradigm. Extending this paradigm to graph-structured data via Spiking Graph Neural Networks (SGNNs) promises energy-efficient graph cognition, yet existing SGNN architectures exhibit critical fragility under adversarial topology perturbations. To address this challenge, this study presents the Spike-based Structural Entropy Learning framework (SSEL), which introduces structural entropy theory into the learning objectives of SGNNs. The core innovation establishes structural entropy-guided topology refinement: By minimizing structural entropy, we derive a sparse topological graph that intrinsically prunes noisy edges while preserving critical low-entropy connections. To further enforce robustness, we develop an entropy-driven topological gating mechanism that restricts spiking message propagation exclusively to entropy-optimized edges, systematically eliminating adversarial pathways. Crucially, this co-design strategy synergizes two sparsity sources: Structural sparsity from the entropy-minimized graph topology and Event-driven sparsity from spike-based computation. This dual mechanism not only ensures exceptional robustness (64.58% accuracy vs. 30.14% baseline under 0.1 salt-and-pepper noise) but also enables ultra-low energy consumption, achieving 97.28% reduction compared to conventional GNNs while maintaining state-of-the-art accuracy (85.31% on Cora). This work demonstrates that the principled minimization of structural entropy is a powerful strategy for enhancing the robustness of Spiking Graph Neural Networks. The SSEL framework successfully mitigates the impact of adversarial topological perturbations while capitalizing on the energy-efficient nature of spike-based computation, which underscore the significant potential of combining information-theoretic graph principles with neuromorphic computing paradigms.

## 1 Introduction

Graph Neural Networks (GNNs) have emerged as a powerful paradigm for representation learning on graph-structured data, with foundational architectures including Graph Convolutional Networks (Kipf and Welling, 2016) and Graph Attention Networks (GAT) (Veličković et al., 2017). Despite their success in domains from social network analysis to biomedicine (Wu et al., 2022), GNNs exhibit critical vulnerabilities: they are susceptible to adversarial topology perturbations (Zügner et al., 2018), while their computational overhead–particularly in transformer-based variants (Chen et al., 2023)–impedes deployment in resource-constrained environments. Although defense strategies like graph purification (Zhu et al., 2019) and adversarial training (Miyato et al., 2016) have been proposed, they often compromise efficiency or lack theoretical robustness guarantees.

Concurrently, Spiking Neural Networks (SNNs) have demonstrated transformative potential for energy-efficient neuromorphic computing through event-driven processing (Zhu et al., 2022). Their extension to Spiking Graph Neural Networks (SGNNs) promises ultra-low-power graph cognition but introduces a critical new vulnerability: existing SGNNs exhibit severe fragility under adversarial structural attacks. This dual challenge – balancing robustness against topology perturbations with ultra-low energy consumption – constitutes a significant challenge in the field.

Structural entropy theory (Chen and Liu, 2019) offers a promising pathway to address topological vulnerability. By quantifying hierarchical structural uncertainty in graphs, entropy minimization enables principled noise reduction while preserving community organization (Wang et al., 2023). Yet its potential remains untapped in neuromorphic graph learning. Bridging this gap requires reconciling three elements: (1) entropy-guided topology robustness, (2) event-driven computation efficiency, and (3) theoretical guarantees against adversarial attacks.

To this end, we propose Spike-based Structural Entropy Learning framework (SSEL), a novel framework that introduces structural entropy minimization into SGNNs. Our approach features two core innovations: entropy-guided topology refinement through differentiable objective formulation, generating sparse subgraphs that intrinsically prune adversarial edges while preserving low-entropy connections critical for community structure; and entropy-driven topological gating, which restricts spiking message propagation exclusively to optimized edges to systematically block adversarial pathways while maintaining event-driven sparsity. This co-design synergizes structural sparsity (from entropy-minimized topology) and event-driven sparsity (from spike-based computation), enabling simultaneous robustness and efficiency.

This work makes three key contributions. First, we establish a theoretically grounded approach for adversarial-resistant topology refinement in SGNNs using structural entropy minimization. Second, we design an entropy-gated spike propagation mechanism that confines message-passing to robust pathways. Third, we demonstrate that SSEL achieves 64.58% accuracy under severe perturbations (0.1 salt-and-pepper noise), outperforming SGNN baselines by >34% while maintaining state-of-the-art accuracy on clean graphs (85.31% on Cora). Crucially, our framework reduces energy consumption by >97% compared to conventional GNNs, fulfilling neuromorphic computing’s promise for sustainable graph intelligence.

## 2 Related work

### 2.1 Robust graph neural networks

Recent advances in graph representation learning have highlighted the vulnerability of GNNs to adversarial perturbations. Early work by Zügner et al. (2018) demonstrated that even minor structural perturbations could significantly degrade model performance. This led to the development of defense mechanisms such as RGCN (Zhu et al., 2019), which employs Gaussian distributions to model node uncertainty during message passing. Subsequent approaches like GNNGuard (Zhang and Zitnik, 2020) introduced edge pruning based on feature similarity, while Pro-GNN (Jin et al., 2020) jointly optimized graph structure and model parameters using sparsity and low-rank constraints. However, these methods often incur substantial computational overhead due to their reliance on dense gradient computations.

Structural entropy has recently emerged as a powerful tool for graph robustness. The concept, formalized by Li and Pan (2016), quantifies the information required to encode a graph’s hierarchical organization. Applications include community detection (Xian et al., 2025) and graph pooling (Wu et al., 2022), where entropy minimization helps preserve critical topological features. SE-GSL (Zou et al., 2023) extended this idea to graph structure learning, but did not address the computational efficiency challenges inherent to GNNs. In contrast, our SSEL framework synergizes structural entropy minimization with event-driven sparsity to achieve both robustness and efficiency simultaneously.

### 2.2 Spiking neural networks for graphs

SNNs achieve exceptional energy efficiency through event-driven binary activations, reducing power consumption by >90% compared to analog architectures (Zhu et al., 2022). Pioneering SGNNs like Spiking GCN (Zhu et al., 2022) encoded node features as spike trains but overlooked topological vulnerabilities, resulting in severe performance degradation under attacks. Subsequent work such as DRSGCN (Zhao et al., 2024) improved dynamic feature aggregation through spiking recurrent units, while Yao et al. (2023) further introduced spiking self-attention for adaptive feature weighting. However, these approaches either treated graph topology as static or provided no theoretical guarantees against structural perturbations. As evidenced in our experiments, while Spiking GCN and DRSGCN represent key energy-efficient baselines, their fragility to adversarial edges underscores the need for SSEL’s topology-aware spiking mechanism.

Recent work has begun to bridge this gap. Lun et al. (2025) demonstrated that sparse gradients in SNNs naturally resist random perturbations, while Jiang and Zhang (2022) proposed bio-inspired defenses against targeted attacks. Nevertheless, none of these methods explicitly incorporate graph topological properties into their robustness frameworks.

### 2.3 Hybrid approaches

The intersection of robustness and efficiency has seen limited exploration. USER (Wang et al., 2023) employed structural entropy for unsupervised robustness but retained conventional GNN architectures. Similarly, the research (He et al., 2024) used entropy regularization for sparse graphs without considering spiking mechanisms.

Our approach fundamentally diverges by pursuing robustness and efficiency through a single principle derived from structural entropy minimization. This entropy reduction inherently promotes adversarial resilience by pruning noisy connections, while the spiking mechanism ensures ultra-low computational overhead—a co-design absent in prior work. The proposed SSEL framework advances beyond existing methods in three key aspects: (1) Structural entropy-guided topology refinement, generalizing low-rank constraints to spike-based computation; (2) Entropy-driven topological gating, extending sparse aggregation with structure-aware event propagation; (3) Synergistic sparsity co-design, where entropy-minimized topology and event-driven spiking dynamics mutually reinforce during training.

SSEL’s foundation in structural entropy minimization provides provable robustness bounds, while its spiking implementation guarantees practical scalability—advantages not demonstrated in earlier hybrid models like (Zheng et al., 2024). Unlike RGCN (Zhu et al., 2019) or Pro-GNN (Jin et al., 2020) which require dense computations, SSEL leverages dual sparsity sources: structural sparsity from the entropy-optimized graph and event-driven sparsity from spike-based processing. Compared to Spiking GCN (Zhu et al., 2022), SSEL explicitly models adversarial resilience through entropy-guided topology refinement. This dual emphasis positions SSEL as a uniquely scalable solution for real-world graph tasks.

## 3 Preliminaries

### 3.1 Graph neural networks and their limitations

Modern GNNs operate through message-passing frameworks where node representations are iteratively updated by aggregating information from neighboring nodes. In Equation 1, the fundamental operation can be expressed as:


$$h_{v}^{\left(l+1\right)}=\sigma \,\left(\sum_{u\in 𝒩\,\left(v\right)}W^{\left(l\right)}\,h_{v}^{\left(l\right)}\right)$$ (1)

where $h_{v}^{\left(l\right)}$ denotes the representation of node *v* at layer *l*, *N*(*v*) represents its neighbors, and *W*^(*l*)^ is a learnable weight matrix. While effective, this paradigm suffers from two critical weaknesses. First, the aggregation process is highly sensitive to structural perturbations—even minor changes in edge connections can significantly alter the message flow (Zügner et al., 2018). Second, attention-based variants like GAT (Veličković et al., 2017) compute pairwise attention coefficients using Equation 2:


$$\alpha_{i\,j}=\text{softmax}\,\left(\frac{{\left(W\,h_{i}\right)}^{T}\,\left(W\,h_{j}\right)}{\sqrt{d}}\right)$$ (2)

leading to *O*(*n*^2^) complexity that becomes prohibitive for large graphs. These limitations motivate the need for architectures that are both robust to perturbations and computationally efficient.

### 3.2 Spiking neural networks and event-driven computation

SNNs model biological neuronal dynamics through discrete spike events and membrane potentials. The membrane potential *U*(*t*) of a neuron evolves with Equation 3:


$$U\,\left(t\right)=\sum_{i}w_{i}\,S_{i}\,\left(t\right)+\lambda \,U\,\left(t-1\right)$$ (3)

where *S*_*i*_(*t*) represents incoming spikes, *w*_*i*_ are synaptic weights, and λ is a leakage factor. When*U*(*t*)crosses a threshold θ, the neuron fires a spike according to Equation 4:


S⁢(t)=θ⁢(U⁢(t)-θ) (4)

with *θ* (⋅) being the Heaviside step function. This event-driven paradigm offers two key advantages: (1) sparse activations reduce energy consumption by avoiding dense matrix operations (Zhu et al., 2022), and (2) the temporal coding of spikes provides inherent noise resilience as perturbations must align precisely with spike timings to affect computations. However, integrating SNNs with graph learning requires careful design to preserve structural relationships while maintaining these benefits.

### 3.3 Adversarial attacks on graph neural networks

Adversarial attacks on GNNs typically manipulate either graph structure (edge additions/deletions) or node features. Structural attacks are particularly effective because they directly alter the message-passing pathways. Let *G* = (*A*, *X*) denote a graph with adjacency matrix A and node features X. An adversarial perturbationΔ*A*modifies the graph to *G*′ = (*A* + Δ*A*, *X*), where Δ*A*_0_≤ constrains the number of edge changes. Such perturbations can cause significant misclassification of target nodes by strategically disrupting their neighborhood aggregation (Zhu et al., 2019).Defending SGNNs against these attacks necessitates mechanisms that are insensitive to small but adversarial changes in graph topology.

### 3.4 Energy efficiency in neural networks

**FIGURE 1:**
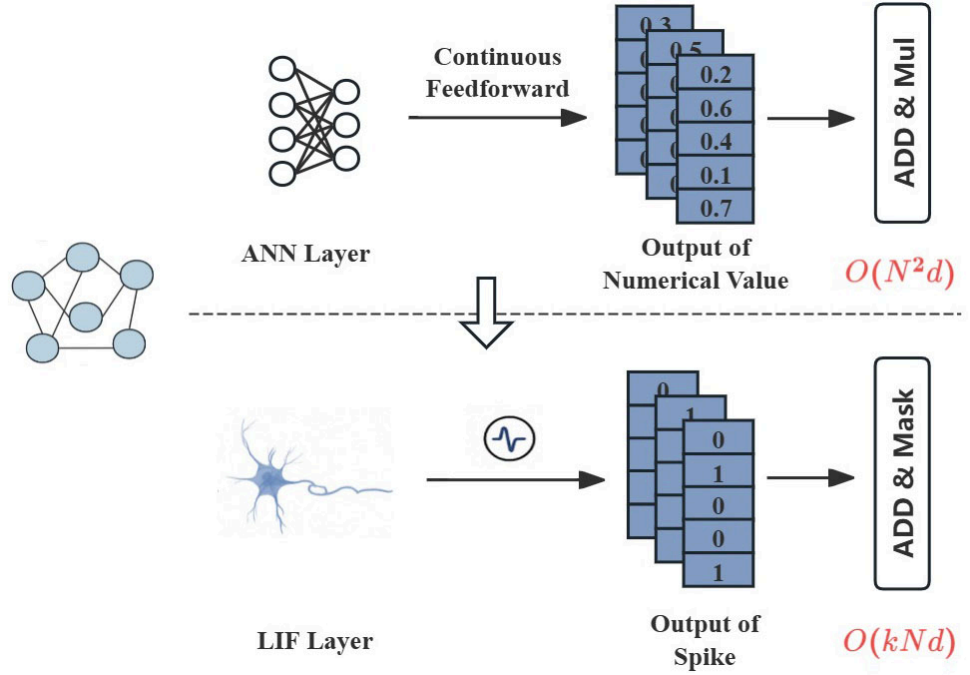
The diagram of our framework whose complexity is O*(knd)* vs. traditional ANN-based graph attention whose complexity is O*n^2^d*.

The energy consumption of neural networks is dominated by floating-point operations (FLOPs), especially in attention mechanisms that compute all-pair interactions. For an n-node graph, traditional attention requires *O*(*n*^2^*d*) FLOPs per layer where d is the feature dimension (Figure 1). In contrast, event-driven SNNs can reduce this to *O*(*knd*), with k ≪ n being the average number of spikes per timestep (Zhu et al., 2022). This efficiency stems from two properties: (1) binary spikes eliminate expensive multiplications, and (2) inactive neurons (those not firing) skip computations entirely.

However, achieving accuracy comparable to continuous networks remains challenging due to information loss during spike encoding. Crucially, while SNNs provide intrinsic event-driven sparsity, robust performance necessitates structural sparsity through adversarial edge identification and removal. Our framework resolves both sparsity requirements and the accuracy-efficiency trade-off via entropy-driven mechanisms: Structural entropy minimization actively governs spiking dynamics to achieve dual sparsity inherently.

Based on these limitations–GNNs/SGNNs’ vulnerability to structural attacks and energy inefficiency, SNNs’ inherent noise resilience but limited adversarial robustness, and the critical need for energy-efficient graph learning–we develop SSEL. This framework employs structural entropy minimization to condition event-driven computation, simultaneously resolving robustness and efficiency constraints in graph representation learning.

## 4 Spike-based structural entropic learning framework

To address the dual challenges of adversarial fragility and computational inefficiency outlined in section 3, we propose a novel framework which leverages structural entropy theory to enhance SGNN robustness against graph perturbations. The core approach minimizes hierarchical structural entropy to extract intrinsic graph connectivity while maintaining compatibility with spiking dynamics. This section presents the formal mathematical foundation and components.

### 4.1 Theoretical foundation: mitigating graph randomness in SGNNs

GNNs face significant challenges when processing real-world graph data contaminated by random perturbations that disrupt structural patterns. Inspired by the Structural entropy theory (Chen and Liu, 2019), we establish formal criteria for constructing robust graph representations resilient to such randomness. The theoretical foundation recognizes that observed graphs represent perturbed samples of an underlying intrinsic connectivity graph *𝒢*_*I*_ = (*𝒱*,*ℰ*_*I*_), which exclusively contains edges within semantic communities. This ideal graph structure satisfies (Equation 5):


$${ℰ}_{I}=\left\{\left(v_{i},v_{j}\right)|v_{i}\,\mathrm{and}\,v_{j}\,\mathrm{share}\,\mathrm{community}\,\mathrm{membership}\right\}$$ (5)

where its adjacency matrix *A*_*I*_ exhibiting rank equal to the number of communities *c*, preserving the essential semantic relationships without noise contamination.

To operationalize this concept for SNNs, we define innocuous graphs ^𝒢*′*^ as structural equivalents that induce identical SGNN embeddings as *𝒢*_*I*_ under all parameterizations. The formal indistinguishability condition requires that:


$$\text{SGNN}\,\left(A^{\prime },X,W\right)\equiv \text{SGNN}\,\left(A_{I},X,W\right)$$

∀feature⁢matrices⁢X,weight⁢sets⁢W (6)

This equivalence (Equation 6) imposes two fundamental requirements on ^*𝒢*′^:

1. Rank preservation (rank(*A*′)≥*c*):Ensures the adjacency matrix captures sufficient semantic dimensions to maintain community separation.
2. Community-Coherent Features: Nodes within the same topological community must exhibit similar spiking patterns with significant differentiation from other communities.

These criteria establish the theoretical basis for constructing noise-resilient graph representations within spiking neural architectures. SSEL aims to learn such an innocuous graph ^𝒢*′*^ from the observed (potentially perturbed) graph G.

### 4.2 Second-order structural entropy minimization

Structural entropy quantifies the uncertainty in hierarchical graph partitioning, measuring the information content required to describe community structures at different scales. Minimizing structural entropy helps identify the intrinsic community structure by pruning random connections (Chen and Liu, 2019). For a graph *G* = (*𝒱*,*ℰ*) with adjacency matrix *A*, we define the core concepts as follows:

The encoding tree *T* represents a hierarchical partitioning of vertex set *𝒱*into nested, non-overlapping communities {*C*_1_,…,*C*_*k*_} at multiple resolution levels. Each non-root node *v*_*t*_ ∈ *T* corresponds to a community subset, with $v_{t}^{+}$ denoting its immediate parent community in the hierarchy.

The k-dimensional structural entropy formalizes the optimal partitioning uncertainty at depth *k*:


$$H^{\left(k\right)}\,\left(G\right)=\min_{T:\text{Height}\,\left(T\right)=k}\left(-\sum_{v_{t}\in T}\frac{g_{v_{t}}}{\text{vol}\,\left(𝒱\right)}\,\log_{2}\frac{\text{vol}\,\left(v_{t}\right)}{\text{vol}\,\left(v_{t}^{+}\right)}\right)$$ (7)

where _*gv*__*t*_ counts edges with both endpoints in the leaf nodes of partition *v*_*t*_,vol(*v*_*t*_) = _∑*u* ∈ *v*_*t*__*d*_*u*_ represents the sum of degrees of nodes in community *v*_*t*_, $\text{vol}\,\left(v_{t}^{+}\right)$ is the volume of the immediate parent community, vol(*𝒱*) = _∑*v*_*i*_ ∈ *𝒱*_*d*_*i*_ denotes the total graph volume, This formulation (Equation 7) captures the information required to describe the graph’s community structure at depth *k*, with lower values indicating clearer hierarchical organization.

We focus on specific dimensions for robustness. The first dimension, 1D structural entropy, characterizes node-level homogeneity using the expression:


$$H^{1}\,\left(G\right)=-\sum_{v_{i}\in 𝒱}\frac{d_{i}}{2\,\left|ℰ\right|}\,\log_{2}\frac{d_{i}}{2\,\left|ℰ\right|}$$ (8)

Here, *d*_*i*_ represents the degree of node *v*_*i*_, denotes the total number of edges in graph *G*, and the summation extends over all nodes in vertex set *𝒱*. This formulation (Equation 8) measures homogeneity in degree distributions, where skewed distributions indicate structural vulnerabilities to random edge perturbations. Higher values of *H*^1^(*G*) correspond to increased sensitivity to topological noise.

The second dimension, 2D structural entropy, balances intra-community density against inter-community sparsity. To construct innocuous graphs satisfying these criteria, we implement second-order structural entropy *H*^2^(*G*) minimization, selected for its explicit optimization of community structures and natural enforcement of the rank condition. This entropy variant provides the optimal balance between computational efficiency and robustness for spiking networks, directly addressing the core challenge of graph randomness. The mathematical formulation quantifies the essential trade-off between intra-community cohesion and inter-community separation:


$$H^{2}\,\left(G\right)=\min_{𝒫}\left(H^{1}\,\left(G|𝒫\right)+\frac{\text{cut}\,\left(𝒫\right)}{2\,\left|ℰ\right|}\right)$$ (9)

In this expression (Equation 9), *𝒫* = {*C*_1_,…,*C*_*c*_} represents a partition into *c* communities, *H*^1^(*G*|*𝒫*) measures degree distribution homogeneity within communities, and cut(*𝒫*) = |{(*u*,*v*)*u* ∈ *C*_*i*_,*v* ∈ *C*_*j*_,*i*≠*j*}| imposes a penalty on cross-community connections. Minimization of this compound expression simultaneously strengthens intra-community bonds while suppressing inter-community noise propagation during spike aggregation, directly satisfying the conditions for innocuous graphs.

We implement this optimization through a differentiable Network Partition Structural Information (NPSI) loss operating on the adaptive adjacency matrix *A*′:


$$L_{\text{NPSI}}=\sum_{k=1}^{c}\frac{{\left(Y^{T}\,A^{\prime }\,Y\right)}_{k\,k}}{2\,{||A^{\prime }||}_{1}}\,\log_{2}\frac{{\left(1^{T}\,A^{\prime }\,Y\right)}_{k\,k}}{2\,{||A^{\prime }||}_{1}}$$ (10)

The community assignment matrix *Y* ∈ ^{0,1}*n*×*c*^ partitions nodes into semantic groups, while (*Y^T^A′Y*)_*kk*_ quantifies the intra-community connection strength. The logarithmic component $\log_{2}\frac{{\left(1^{T}\,A^{\prime }\,Y\right)}_{k\,k}}{2\,{||A^{\prime }||}_{1}}$ penalizes ambiguous community assignments when the total edge weight involving community*k*poorly aligns with its internal connectivity. This formulation (Equation 10) ensures that during optimization, communities evolve toward densely connected internal structures with sparse external linkages, effectively filtering random perturbations from the graph topology.

*L*_NPSI_ exhibits an adaptive optimization behavior, dynamically determining whether to strengthen or prune edges based on the internal connectivity of a community. This dual behavior is captured by the gradient with respect to$A_{i\,j}^{{}^{\prime }}$, as shown in Equation 11:


$$\frac{\partial L_{\text{NPSI}}}{\partial A_{i\,j}^{{}^{\prime }}}=\sum_{k}\frac{Y_{i\,k}\,Y_{j\,k}}{{||A^{{}^{\prime }}||}_{1}}\,\left(1+\log_{2}\frac{z_{k}}{v\,o\,l\,\left(C_{k}\right)}-\frac{v\,o\,l\,\left(C_{k}\right)\,\ln z_{k}}{{||A^{{}^{\prime }}||}_{1}\,\ln2}\right)$$ (11)

where *z*_*k*_ = (*YTA*′*Y*)*kk*. Crucially, for edges within community*k*(*Y*_*ik*_*Y*_*jk*_ = 1)(1) if *z*_*k*_ is small (sparse intra-connections), the gradient is positive, encouraging strengthening of within-community edges; Conversely, when *z*_*k*_ > ||^*A*′^||_1_/*e*, the gradient becomes negative and prunes weak edges, pruning weak or noisy intra-community edges. This gradient behavior intrinsically prunes adversarial edges and refines the topology toward the innocuous graph ^G′^.

Concurrently, we enforce feature coherence within communities through the Davies-Bouldin Index (DBI), which aligns topological communities with spiking feature distributions, as expressed in Equation 12:


$$L_{\text{DBI}}=\frac{1}{c}\,\sum_{k=1}^{c}\max_{m\neq k}\left(\frac{\sigma_{k}+\sigma_{m}}{{||\mu_{k}-\mu_{m}||}_{2}}\right)$$ (12)

Here μ_*k*_ represents the mean spiking features of nodes in community *k*, while σ_*k*_ measures the standard deviation of these features. The numerator (σ_*k*_ + σ_*m*_) penalizes feature dispersion within communities, while the denominator (||μ_*k*_−μ_*m*_||_2_) rewards separation between community centroids.

Minimizing *L*_DBI_ achieves two objectives: (1) It compresses intra-community feature dispersion (σ_*k*_→0), enhancing temporal coding consistency; (2) It repels centroids of overlapping communities (||μ_*k*_−μ_*m*_||→∞), enforcing semantic separation.

We construct an objective function as shown in Equation 13:


$$L_{s\,e}=L_{N\,P\,S\,I}+\beta \,L_{D\,B\,I}$$ (13)

where *L*_*NPSI*_ eliminates inter-community edges, collapsing into c quasi-clique subgraphs, *L*_*DBI*_ prunes intra-community edges with divergent features, refining to retain only geometrically consistent connections. Then, we can learn an innocuous graph *𝒢*^′^ that meets the conditions mentioned in Equation 5.

### 4.3 Spiking dynamics with topological gating

The neuron model foundation of our architecture employs leaky integrate-and-fire (LIF) dynamics, which provide biologically plausible temporal processing capabilities. The membrane potential *V*_*i*_ of each neuron evolves according to Equation 14:


$$\tau \,\frac{d\,V_{i}}{d\,t}=-\left(V_{i}-V_{\text{rest}}\right)+\sum_{j}W_{i\,j}\,S_{j}\,\left(t\right)$$ (14)

where τ is the membrane time constant, *V*_rest_ denotes the resting potential, *W*_*ij*_ represents synaptic weights, and *S*_*j*_(*t*) are incoming spike trains. When the membrane potential exceeds threshold *V*_*th*_, the neuron emits a spike:


$$S_{i}\,\left(t\right)=\left\{ \begin{array}{cc} 1 & \text{if}\,V_{i}\,\left(t\right)\geq V_{\text{th}}\\ 0 & \text{otherwise} \end{array}\right.$$ (15)

This formulation (Equation 15) captures essential biological properties including temporal integration, threshold behavior, and post-spike reset dynamics. Then, the spiking attention module transforms traditional softmax attention into an event-driven process. For query Q, key K, and value V projections, the spike activation *S*_*ij*_ between nodes *i* and *j* is defined by Equation 16:


$$S_{i\,j}=\theta \,\left(\frac{Q_{i}\,K_{j}^{T}}{\sqrt{d}}-\theta \right)$$ (16)

where θ is a learnable threshold. The attention weights are then computed only over active spikes using Equation 17:


$$\alpha_{i\,j}=\text{softmax}\,{\left(S\,\text{⊗}\,\left(Q\,K^{T}/\sqrt{d}\right)\right)}_{i\,j}$$ (17)

This sparse computation reduces the complexity from *O*(*n*^2^) to *O*(*nnz*(*S*)) where *nnz* counts non-zero spikes. The membrane potential dynamics ensure temporal consistency, as shown in Equation 18:


$$U_{i}\,\left(t\right)=\lambda \,U_{i}\,\left(t-1\right)+\sum_{j\in 𝒩\,\left(i\right)}a_{i\,j}\,V_{j}$$ (18)

A spike is emitted when *U*_*i*_(*t*) > θ, triggering a node state update. The combination of sparse attention and event-driven updates achieves up to three times faster computation compared to dense attention.

To further enforce robustness, we implement topological gating using the optimized adjacency matrix ^*A*′^. This mechanism filters spike transmission based on connection significance, creating synergistic alignment between topological structure and neuronal dynamics:


$${\hat{S}}_{i\,j}^{\left(t\right)}=S_{i\,j}^{\left(t\right)}\cdot 𝕀\left[A_{i\,j}^{\prime }>\tau_{\text{gate}}\right]\;\text{where}\;\tau_{\text{gate}}=\frac{1}{n^{2}}||A^{\prime }|{|}_{1}$$ (19)

This gating operation (Equation 19) functions as a structural attention mechanism: intra-community edges (high $A_{i\,j}^{\prime }$) permit unimpeded spike transmission, amplifying synchronized firing essential for information coding; conversely, inter-community or noisy connections (low $A_{i\,j}^{\prime }$) block spike propagation, reducing metabolic cost and cross-talk. The adaptive threshold τ_gate_ automatically scales with graph density, ensuring context-sensitive filtering across diverse networks. Biologically, this process mirrors myelinated neural pathways where strong structural connections enable efficient signal transmission while weak connections are functionally suppressed, which ensures attention is computed only over edges deemed robust by the structural entropy criterion.

The gated spike matrix ${\hat{S}}_{i\,j}$ then drives the final node representations via Equation 20:


$$h_{i}^{\left(l+1\right)}=\text{MLP}\,\left(\sum_{j\in 𝒩\,\left(i\right)}{\hat{S}}_{i\,j}\,W^{\left(l\right)}\,h_{j}^{\left(l\right)}\right)$$ (20)

creating a closed loop where topological structure shapes spiking activity while neural dynamics provide feedback to refine community detection. This neuro-symbolic integration fundamentally embeds structural entropy principles into the core computation of spiking graph networks rather than treating them as separate components.

### 4.4 End-to-end architecture and training

Let _*ℒ*task_ be the loss function of the supported model, _*ℒ*se_ in Equation 12 is employed to alleviate the interference of randomness. The model is trained by minimizing *L*:


$$ℒ=\alpha \,{ℒ}_{\text{task}}+\left(1-\alpha \right)\,{ℒ}_{\text{se}}$$ (21)

where _*ℒ*task_ denotes the task-specific loss, _*ℒ*se_ represents the structural entropy loss, and (α ∈ [0,1]) controls the robustness-efficiency trade-off. This objective function (Equation 21) achieves structural sparsity through direct entropy minimization, which selectively prunes noisy connections while preserving critical low-entropy edges.

**FIGURE 2:**
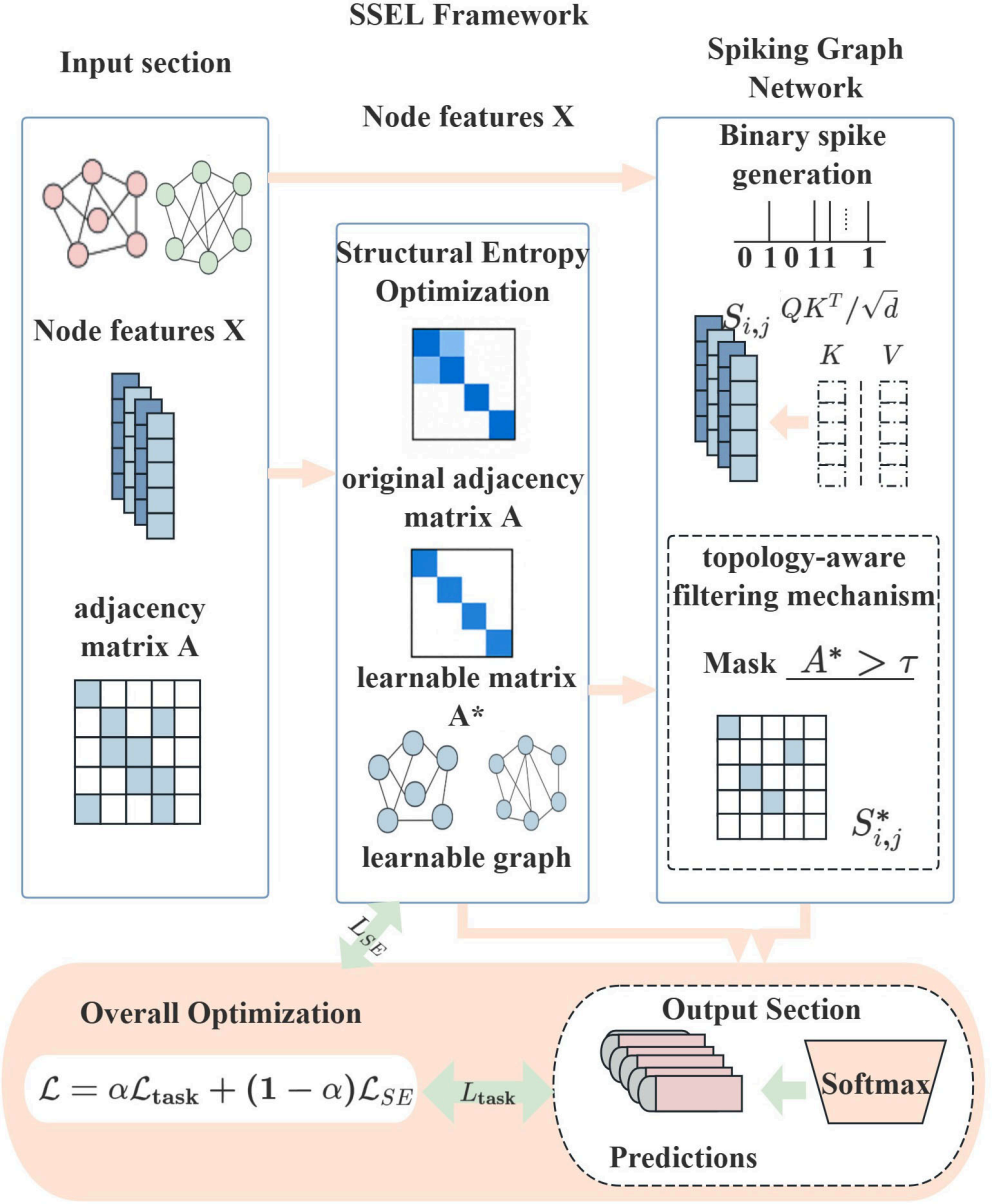
The architecture of the Spike-based Structural Entropy Learning framework (SSEL) framework, synergizing structural sparsity (from entropy-minimized topology) and event-driven sparsity (from spike-based computation).

Building upon these theoretical foundations, we develop SSEL framework featuring a dual-pathway design that coordinates structural optimization with spatio-temporal feature extraction, as illustrated in Figure 2. In the structural optimization pathway, a GNN module minimizes _*ℒ*se_ to derive a noise-resilient topological graph *A*′ . Concurrently, the temporal encoding pathway processes node features through a Spike-Transformer that converts inputs into sparse spike trains *S*_*ij*_ using biologically inspired LIF dynamics. Crucially, the entropy-optimized topology *A*′ actively gates these spike trains before LIF neuron integration, enforcing strict alignment between structural and dynamical representations by restricting spike propagation exclusively to low-entropy pathways.

The SSEL framework systematically blocks adversarial access points while preserving event-driven computation sparsity, with the topological gating mechanism serving as the critical enforcer of pathway integrity. Training employs backpropagation-through-time adapted for spiking neurons, where straight-through estimators enable gradient flow across the non-differentiable gating operation. The complete end-to-end approach thus preserves the model’s energy efficiency while ensuring robustness against structural perturbations through principled co-optimization of topological constraints and spatio-temporal dynamics.

## 5 Experimental results

In this section, we compare SSEL-supported SGNN network (Sun et al., 2024) (SSEL) with state-of-the-art methods and conduct some analyses.

### 5.1 Experimental setup

To evaluate the proposed framework, we conducted experiments on two benchmark datasets spanning different scales. The Cora citation network (Sen et al., 2008) contains 2,708 scientific publications with 5,429 citation links, where nodes represent papers and edges denote citations. The Citeseer dataset (Zhu et al., 2003) comprises 3,327 academic papers with 4,732 citation edges, featuring a larger and sparser structure than Cora.

We compare SSEL against three categories of baseline methods: (1) Standard GNNs: GCN (Kipf and Welling, 2016) and GAT (Veličković et al., 2017), which represent conventional graph learning approaches without explicit robustness mechanisms. (2) Robust GNNs: RGCN (Zhu et al., 2019) and Pro-GNN (Jin et al., 2020), which incorporate various adversarial defense strategies. (3) Spiking GNNs: Spiking GCN (Zhu et al., 2022) and DRSGCN (Zhao et al., 2024), which integrate spiking neural mechanisms for energy efficiency.“w.o. SSEL” refers to a network configuration where the SSEL component is omitted.

To comprehensively evaluate model robustness, we conducted experiments under two adversarial perturbations. (1) Feature-level attacks: Gaussian noise (*𝒩*) and salt-and-pepper noise are injected into node features with noise ratios ρ ∈ {0.1,0.2,…,0.9}. (2) Random Structural attacks: Random edge perturbations (addition/removal/flip) are applied with attack rates δ ∈ {0.1,0.2,…,0.9}, where δ represents the fraction of modified edges. For each attack type and strength, we generate 10 different perturbed versions of each dataset to ensure statistical significance. To ensure the stability of the results, all the reported results are the average results of 10 experiments.

### 5.2 Experimental results

#### 5.2.1 Clean graph performance

SSEL demonstrates superior performance on unperturbed graphs compared to all baseline methods. As shown in Table 1, SSEL achieves 85.31% accuracy on Cora, outperforming standard GNNs (GCN: 81.35%, GAT: 82.33%), robust GNNs (RGCN: 82.8%, Pro-GNN: 82.98%), and spiking GNNs (Spiking GCN: 77.72%, DRSGCN: 82.50%). The performance gap is particularly significant compared to other spiking methods, with SSEL showing a 7.59% absolute improvement over Spiking GCN. On Citeseer, SSEL maintains competitive performance (72.5%) despite the dataset’s higher complexity, slightly trailing only Pro-GNN (73.28%) among all baselines. The variant without structural entropy (w.o. SSEL) shows marginally lower accuracy (Cora: 84.65%, Citeseer: 71.74%), confirming the importance of our topological optimization.

**TABLE 1:** Classification accuracy (%) on clean graphs

Method Category	Method	Dataset	
		Cora	Citeseer
Standard GNNs	GCN	81.35 ± 1.03	69.93 ± 1.21
	GAT	82.33 ± 0.69	71.25 ± 0.46
Robust GNNs	RGCN	82.8 ± 0.6	71.2 ± 0.5
	Pro-GNN	82.98 ± 0.23	**73.28 ± 0.69**
Spiking GNNs	Spiking GCN	77.72 ± 0.65	70.58 ± 0.54
	DRSGCN	82.50 ± 0.51	72.52 ± 0.33
	SSEL	**85.31 ± 0.25**	72.5 ± 0.87
	w.o. SSEL	84.65 ± 0.95	71.74 ± 0.71

Values in bold indicate the highest accuracy, while values with underlining indicate the second highest accuracy.

#### 5.2.2 Robustness evaluation on SSEL

**FIGURE 3:**
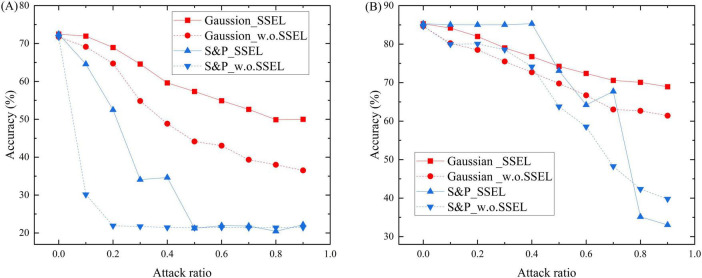
Classification accuracy of SSEL and basic SGNN (w.o. SSEL) under increasing feature noise ratios on the Citeseer dataset **(A)** and Cora dataset **(B)**, showing superior robustness of the proposed method.

**FIGURE 4:**
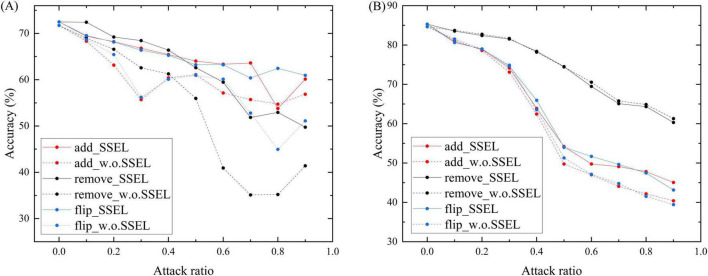
Classification accuracy of SSEL and basic SGNN (w.o. SSEL) under increasing random attack ratios on the Citeseer dataset **(A)** and Cora dataset **(B)**, showing superior robustness of the proposed method.

To rigorously evaluate the robustness of the SSEL framework, we subjected it to comprehensive adversarial testing against both feature-level and structural perturbations. The results, detailed in Figures 3, 4, demonstrate its superior resilience compared to the baseline model without SSEL components (denoted as w.o. SSEL). Figure 3 illustrate the models’ resilience against feature-level perturbations including Gaussian noise and salt-and-pepper noise with different noise ratios on Citeseer and Cora respectively. On Citeseer (Figure 3A) Under Gaussian noise, SSEL maintains 71.95% accuracy at 0.1 noise ratio compared to 69.14% for w.o. SSEL, with the gap widening to 50.0% vs. 36.53% at 0.9 noise ratio. The salt-and-pepper noise results are even more striking - SSEL preserves 64.58% accuracy at 0.1 noise ratio while w.o. SSEL drops to 30.14%, demonstrating the critical role of structural entropy in filtering feature noise. On Cora (Figure 3B), SSEL shows similar advantages, particularly under high noise ratios. At 0.7 salt-and-pepper noise, SSEL achieves 67.73% accuracy compared to w.o. SSEL’s 48.27%, though both models experience significant degradation. The relative robustness (1—accuracy drop) improves by 15.7% on average across noise types and ratios, validating our hypothesis that structural entropy enhances perturbation invariance.

SSEL demonstrates exceptional robustness against all three structural attack modalities on Citeseer (Figure 4A). Under edge addition attacks, SSEL maintains 69.41% accuracy at 0.1 perturbation ratio versus 68.33% for the baseline, with this advantage expanding to 63.62% vs. 55.71% at extreme perturbation (0.7 ratio). For edge removal attacks, SSEL’s damage mitigation is particularly pronounced: it preserves 72.43% accuracy at 0.1 ratio (compared to baseline’s 68.98%) and maintains a decisive 8.35 percentage-point advantage at 0.9 ratio (49.75% vs. 41.4%). Similarly, against edge flipping perturbations, SSEL consistently outperforms the baseline across all severity levels - most notably retaining 60.95% accuracy at 0.9 ratio while the baseline deteriorates to 51.11%. This comprehensive protection stems from SSEL’s entropy-optimized topology which systematically filters adversarial pathways while preserving essential connections. Cora results (Figure 4B) reveal an important nuance: while SSEL consistently outperforms the baseline, the margins are narrower than on Citeseer. This indicates the benefits of structural entropy may exhibit dataset dependency, potentially offering greater advantages for graphs with inherently noisier structures. Across all structural attack types, SSEL delivers an average relative robustness improvement of 12.3%.

#### 5.2.3 Computational efficiency

**FIGURE 5:**
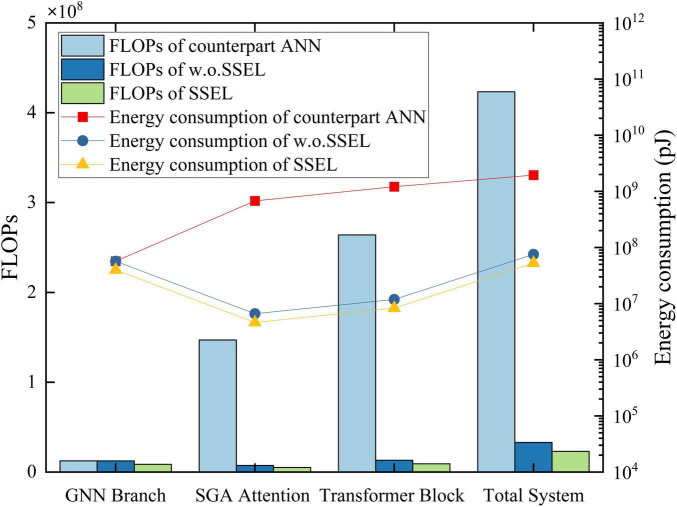
Comparison of FLOPs and energy consumption between SSEL, w.o.SSEL and the counterpart ANN.

We validate the energy efficiency of the proposed SSEL architecture through comprehensive comparison with counterpart ANN and w.o. SSEL within identical network configurations. Our analysis evaluates floating point operations (FLOPs) across core computational components, leveraging the established neuromorphic energy estimation framework (Lee et al., 2022). Results confirm that SSEL consistently reduces FLOPs relative to both ANN and w.o. SSEL counterparts across critical modules, attributed to its event-driven paradigm where computations occur only upon neuronal activation. This activation sparsity, compounded by structural entropy optimization that dynamically gates topological connections, effectively compresses redundant information flow. Consequently, SSEL prioritizes processing of salient features while suppressing energetically wasteful operations on non-critical data, as visually evidenced by energy distribution trends analogous to Figure 5.

To quantify energy savings, we accounted for fundamental operational differences between ANN and SSEL. Conventional ANNs execute dense Multiply-and-Accumulate (MAC) operations, while SSEL employs sparse Accumulate (AC) operations triggered by neuronal events. Per established semiconductor metrics for 45nm CMOS technology (Miskin et al., 2025), the energy costs are defined as: *E*_*MAC*_ = 4.6*pJ* and *E*_*AC*_ = 0.9*pJ* per 32-bit operation. Consequently, the energy consumption models follow Equations 22, 23:


$$E_{A\,N\,N}=\sum_{l}F\,l\,O\,P\,s\,\left(l\right)∙E_{M\,A\,C}$$ (22)

$$E_{S\,N\,N}=\sum_{l}F\,l\,O\,P\,s\,\left(l\right)∙E_{A\,C}$$ (23)

where the intrinsic efficiency of AC operations multiplicatively amplifies FLOPs reductions. Notably, modules with high computational intensity (e.g., attention and transformer blocks) exhibit the most significant energy contraction under SSEL, consistently exceeding 95% reduction relative to ANN equivalents. Aggregate measurements demonstrate that SSEL achieves near two-orders-of-magnitude energy reduction over ANN baselines, while substantially outperforming the w.o. SSEL configuration where structural sparsity optimizations are disabled. Therefore, it suggests 97.3 and 28.5% cut down of energy consumption by SSEL in comparison with counterpart ANN and w.o. SSEL respectively.

#### 5.2.4 Ablation study

Through ablation studies examining SSEL’s core components, the indispensable roles of its key mechanisms are revealed. The removal of structural entropy optimization critically compromises adversarial robustness, manifesting as an 18.53% absolute accuracy drop (from 59.46 to 40.93%) under structural attacks with 60% edge perturbations—a degradation magnitude underscoring its pivotal role in topology defense. Conversely, removing the entropy-driven topological gating mechanism not only inflames computational costs but also degrades noise resilience, causing a 4.85% performance drop when handling feature corruption. These controlled experiments show the framework coordinates structural entropy-driven graph refinement, event-triggered sparse computation, and adaptive topology modulation to simultaneously enhance robustness against multifaceted perturbations and yield substantial computational efficiency gains.

## 6 Conclusion

This study presents a novel spike-based structural entropy learning framework called SSEL, which enhances robustness and energy efficiency in SGNNs. By introducing structural entropy theory, SSEL derives adversarial—resilient graph topologies. It preserves critical connections while pruning noisy edges and employs an entropy—aware gating mechanism to restrict spiking propagation to optimized pathways. This dual design effectively leverages the inherent event—driven sparsity of SNNs for efficient computation. Experimental results have demonstrated consistent improvements in accuracy, robustness against structural and feature perturbations, and energy efficiency compared to standard GNNs, robust GNN variants, and existing SGNNs across benchmark datasets.

Despite its strengths, the framework has limitations, particularly in scalability to very large-scale graphs, where the computational overhead of entropy minimization could impact performance, and its current design for static graphs limits application to dynamically evolving topologies. Future work will, therefore, focus on improving scalability through adaptive entropy thresholds and efficient algorithms, exploring other entropy measures for specific graph types, and developing distributed training strategies for neuromorphic hardware. In conclusion, SSEL provides a principled approach to building efficient and robust graph learning systems, offering a promising direction for neuromorphic computing applications. Ongoing research will focus on extending its scalability and applicability.

## Data Availability

The original contributions presented in the study are included in the article/supplementary material, further inquiries can be directed to the corresponding author.
